# Colonic Dysregulation of Major Metabolic Pathways in Experimental Ulcerative Colitis

**DOI:** 10.3390/metabo14040194

**Published:** 2024-03-29

**Authors:** Ji Yeon Noh, Naser Farhataziz, Michael T. Kinter, Xin Yan, Yuxiang Sun

**Affiliations:** 1 Department of Nutrition, Texas A&M University, College Station, TX 77843, USA; jynoh@tamu.edu (J.Y.N.); naserfarhataziz@tamu.edu (N.F.); 2 Aging and Metabolism Research Program, Oklahoma Medical Research Foundation, Oklahoma City, OK 73104, USA; mike-kinter@omrf.org; 3 Department of Chemistry, Texas A&M University, College Station, TX 77843, USA; xyan@tamu.edu; 4 Department of Biochemistry & Biophysics, Texas A&M University, College Station, TX 77843, USA

**Keywords:** inflammatory bowel disease, ulcerative colitis, proteomics, metabolism, antioxidative defense, oxidative stress, β-oxidation, glycolysis, TCA cycle

## Abstract

Inflammatory bowel disease (IBD) is multifactorial chronic inflammatory disease in the gastrointestinal tract, affecting patients’ quality of life profoundly. The incidence of IBD has been on the rise globally for the last two decades. Because the molecular mechanisms underlying the disease remain not well understood, therapeutic development is significantly impeded. Metabolism is a crucial cellular process to generate the energy needed for an inflammatory response and tissue repair. Comprehensive understanding of the metabolic pathways in IBD would help to unravel the disease pathogenesis/progression and facilitate therapeutic discoveries. Here, we investigated four metabolic pathways altered in experimental colitis. C57BL/6J mice were treated with dextran sulfate sodium (DSS) in drinking water for 7 days to induce experimental ulcerative colitis (UC). We conducted proteomics analysis for the colon samples using LC/MS, to profile key metabolic intermediates. Our findings revealed significant alterations in four major metabolic pathways: antioxidative defense, β-oxidation, glycolysis, and TCA cycle pathways. The energy metabolism by β-oxidation, glycolysis, and TCA cycle pathways were downregulated under UC, together with reduced antioxidative defense pathways. These results reveal metabolic re-programming in intestinal cells under UC, showing dysregulation in all four major metabolic pathways. Our study underscores the importance of metabolic drivers in the pathogenesis of IBD and suggests that the modification of metabolism may serve as a novel diagnostic/therapeutic approach for IBD.

## 1. Introduction

Inflammatory bowel disease (IBD) is a digestive disorder that results from excessive levels of inflammation and tissue damage in the gastrointestinal tract. The incidence of IBD has been growing globally for the last two decades, especially in developed countries [1]. IBD is commonly associated with persistent diarrhea, abdominal pain, rectal bleeding, and weight loss [2,3]. IBD is classified into two types, ulcerative colitis (UC) and Crohn’s disease (CD) [4]. While the symptoms of both UC and CD are similar, the inflammatory sites differ as CD is often accompanied by systematic inflammation in both the small intestine and colon, while UC typically affects the colon [3]. Prolonged inflammation of the bowels can lead to permanent damage and deterioration of the epithelium, resulting in uncontrolled inflammation as the disease progresses [5]. IBD severely impacts patients’ social life, economic status, physical health, and mental health [6,7,8]. In addition, IBD has been associated with colorectal cancer, small bowel adenocarcinoma, intestinal lymphoma, and cholangiocarcinoma [9,10]. Furthermore, IBD is associated with other comorbidities, including cardiovascular, hepatic, and psychiatric disorders, and obesity [11,12]. IBD is a poorly understood disease; its diagnosis is often delayed compared to other diseases [3]. In an international survey, 45% of respondents stated they received a diagnosis more than a year after symptoms appeared, and 17% of respondents stated that their diagnosis was received more than 5 years after the onset of symptoms [7]. The identification of diagnostic biomarkers for timely detection, and the development of effective therapies are particularly crucial, given the challenging nature of IBD.

Metabolism is a fundamental process that governs all biological and pathological activities by supplying energy and facilitating the signaling transduction of macromolecules. In IBD, alterations in energy metabolism have been observed [13,14,15,16]. Though, existing metabolomics studies show discrepancies in the levels of metabolites in blood, urine, and tissue samples in IBD [13,14,17,18]. Understanding the comprehensive profile and regulation of metabolic pathways at the molecular level is crucial for unraveling the complex interplay between metabolism and inflammation, providing valuable insights for diagnostic biomarkers and therapeutic interventions. In the current study, we induced experimental colitis in C57BL/6J male mice using a dextran sulfate sodium (DSS)-induced colitis model, and colon tissue was analyzed using mass spectrometry to assess altered levels of enzymes that regulate four major metabolic signaling cascades: oxidative stress, β-oxidation, glycolysis, and the TCA cycle.

## 2. Materials and Methods

### 2.1. Animal Model

The C57BL/6J background of 4–6-month-old male mice were used in this study. All mice were housed and bred at Texas A&M University’s laboratory animal facility with controlled lighting and temperature (12 h light–dark cycle, 75 ± 1 °F), with free access to a regular diet of Harlan-Teklad 2018X (Harlan-Teklad, Madison, WI, USA), which contains 58% calories from carbohydrates, 24% calories from protein, and 18% calories from fat. Mice were randomly divided into two groups, and then given either ad libitum water (H_2_O) or 2% (*w*/*v*) dextran sulfate sodium (DSS) in water.

### 2.2. DSS-Induced Colitis

Experimental colitis was induced as described before [19], by providing 2% (*w*/*v*) DSS (MP Biomedicals; 36–50 kDa, Santa Anna, CA, USA) in drinking water for 7 days ad libitum. DSS was replaced every 48 h to maintain its potency. At termination, the mice were euthanized under isoflurane anesthesia.

### 2.3. Tissue Collection and Protein Extraction

Colons were excised from each mouse and flushed with ice-cold PBS. They were opened longitudinally and then flash frozen until further processing. Flash frozen tissue strips were then homogenized at a ratio of 1 g of tissue to 10 mL of RIPA buffer (Sigma, St. Louis, MO, USA) with protease inhibitors, PhosSTOP™ (Sigma, St. Louis, MO, USA) and cOmplete™ (Sigma, St. Louis, MO, USA). Homogenates were centrifuged at high speed for 5 min, and the concentration of total protein was measured using the PierceTM BCA protein assay kit (Thermo Fisher Scientific, Waltham, MA, USA) following the manufacturer’s instructions. In brief, 25 µL of sample was mixed with 200 µL of Bradford reagents, then incubated at 37 °C for 30 min in the dark. Then, the absorbance was read at 590 nm wavelength using a CLARIOstar plate reader (BMG Labtech, Cary, NC, USA). Then, supernatants were aliquoted into volumes equivalent to 100 µg of total protein. Samples were stored in −80 °C until they were subjected to mass spectrometry analysis.

### 2.4. Sample Preparation for Mass Spectrometry

The sample proteins were prepared as we have described [20,21]. For each sample, 60 µg of protein was mixed with 1% SDS and 20 μL of BSA internal standard. Samples were mixed and heated, then proteins were precipitated with acetone. Dried protein pellets were reconstituted in Laemmli sample buffer and then run through a short (1.5 cm) 12.5% SDS-PAGE gel (Bio-Rad, Berkeley, CA, USA) for approximately 15 min at 150 V. The gels were fixed and stained with Coomassie blue (GelCode blue, Pierce, Appleton, WI, USA) for visualization. Samples were excised from the gel as entire lanes and then divided into smaller fragments. Gel segments were washed to remove the stain and then reduced with DTT and alkylated with iodoacetamide. The samples were digested overnight with trypsin (Promega, Madison, WI, USA). Sample proteins were then extracted from the gel and evaporated to dry in a SpeedVac. Samples were then reconstituted in 1% acetic acid for further analysis.

### 2.5. Mass Spectrometry Analysis

Analysis was conducted using the TSQ Quantiva triple quadrupole mass spectrometry system (Thermo Fisher Scientific, Waltham, MA, USA) as previously described [20,21]. In brief, the HPLC was an Ultimate 3000 nanoflow system with 10 cm × 75 µm i.d. C18 capillary column (Phenomenex Jupiter, Torrance, CA, USA). A total of 5 µL aliquots were injected and loaded into the column. The column was eluted with a 60 min gradient of acetonitrile in 0.1% formic acid. The mass spectrometry system was operated under the selected reaction monitoring mode. The detection method for each protein measured for 2 ideal peptides, and assays for multiple proteins were grouped together in larger panels. Determination of the integrated peak area of the appropriate chromatographic peaks was completed using the Skyline data analysis program. Responses for each protein were calculated as the geometric mean of the peptide areas, and values were normalized according to the BSA standard.

### 2.6. Statistical Analysis

Statistical analysis of protein abundance was completed using MetaboAnalyst 5.0 “Statistical Analysis [one factor]” module (http://www.metaboanalyst.ca/ accessed on 1 January 2023). Partial least squares discriminant analysis was used to analyze the clustering of samples and evaluate variable importance in projection. Two-sample, two-tailed t tests were used for the evaluation of significance, and then presented as mean ± SEM using GraphPad Prism 9.5.1 (GraphPad Software, La Jolla, CA, USA). Pathway impact analysis was completed using MetaboAnalyst’s Gene Set Enrichment Analysis Algorithm (GSEA). Fisher’s exact test was used for enrichment analysis, and topology was measured using betweenness centrality.

## 3. Results

### 3.1. Antioxidative Defense Pathway Is Downregulated in Colon of Colitis

Oxidative stress is known to contribute to the pathogenesis of various diseases [22]. Oxidative stress exacerbates epithelial barrier damage in IBD through reactive oxygen species (ROS). ROS lead to the alteration of tight junctions and result in increased gut permeability in the epithelium [23]. To elucidate the effect of antioxidative defense capacity in the colon of UC, the proteins involved in the antioxidative stress pathways were tested using LC/MS. We detected a total of 30 proteins associated with the antioxidative defense pathway.

**Figure 1:**
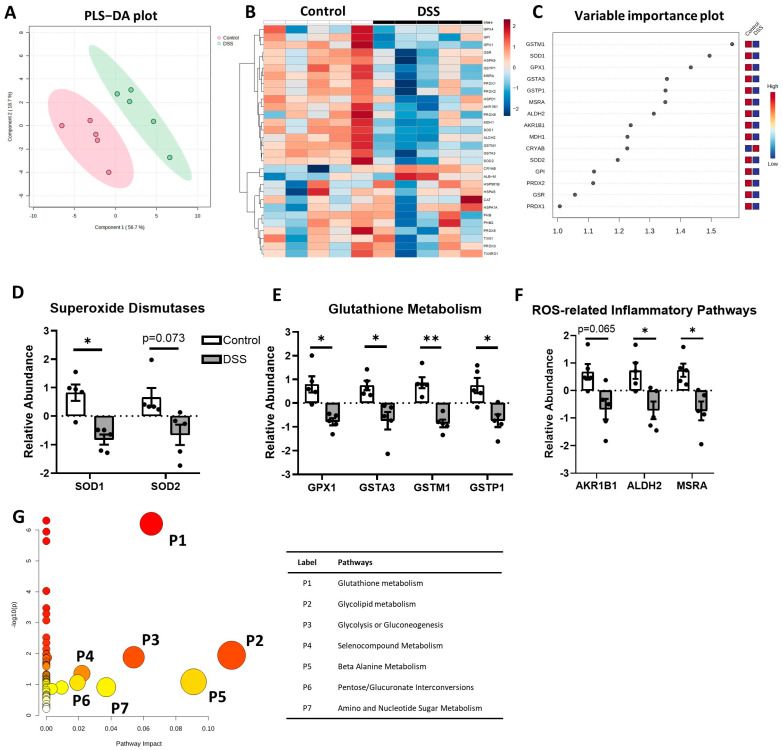
Altered antioxidative stress pathway in DSS-induced colitis. (**A**) Partial least squares discriminant analysis (PLS-DA) biplot; (**B**) non-clustered heatmap of correlating protein concentration with individual samples; (**C**) variable importance plot (VIP) highlighting significantly changed proteins; (**D**) relative abundance of superoxide dismutases (SOD1, SOD2); (**E**) glutathione metabolism-related enzymes (GPX1, GSTA3, GSTM1, GSTP1); (**F**) relative concentrations of ROS-related proteins; (**G**) pathway enrichment impact plot, with color and size of circle indicating pathway impact (larger/more red = higher impact). * *p* < 0.05 and ** *p* < 0.01, DSS vs. Control.

**Figure 2:**
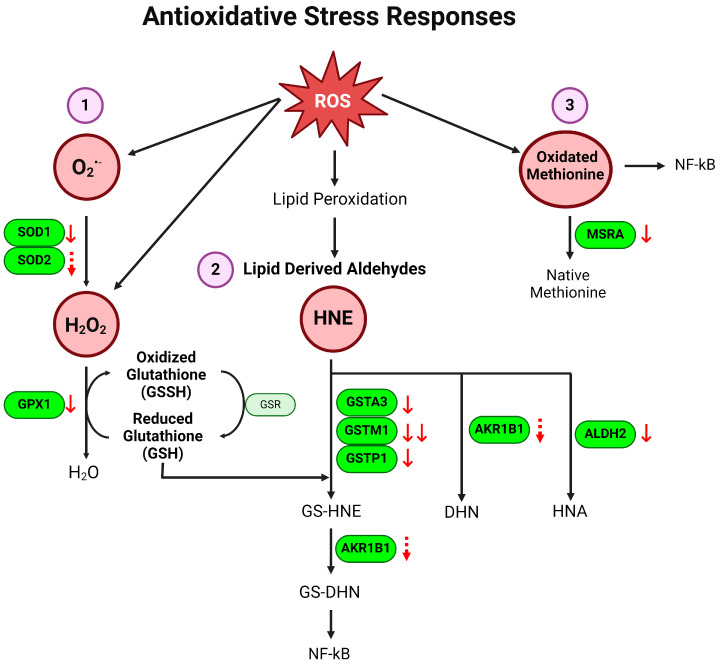
Schematic diagram of downregulated antioxidative responses in DSS-induced colitis. Multiple antioxidative stress-related pathways are altered in colitis. (1) SOD1 and SOD2 convert O_2_^•−^ into H_2_O_2_, then GPX1 oxidizes glutathione to convert H_2_O_2_ into H_2_O. (2) Lipid-derived aldehydes like HNE form complexes with GSH through the GST complex (GSTA3, GSTM1, GSTP1) to form GS-HNE. The GS-HNE is further converted into GS-DHN by AKR1B1. Alternatively, HNE can also be converted to DHN or HNA by AKR1B1 or ALDH2, respectively. (3) ROS-mediated oxidated methionine is converted to native methionine through MSRA. Under colitis, enzymes regulating all three pathways are downregulated as indicated by the red arrows. dotted ↓ *p* < 0.1, ↓ *p* < 0.05, ↓↓ *p* < 0.01, DSS vs. control. SOD: superoxide dismutase; GPX1: glutathione peroxidase; GSH: reduced glutathione; HNE: 4-hydroxynonenal; GST: glutathione S-transferase; GS-HNE: glutathione-HNE; DHN: 1,4-dihydroxy-2-nonene; GS-DHN: glutathione-1,4-dihydroxy-2-nonene; HNA: 4-hydroxy-nonenoic acid; AKR1B1: aldo-keto reductase 1; ALDH2: aldehyde dehydrogenase; MSRA: methionine sulfoxide reductase.

The DSS-treated colitis group and the control group were significantly separated in the partial least squares discriminant analysis (PLS-DA) plot (Figure 1A). The heatmap and variable importance in projection (VIP) score plot revealed that the colitis group had a significantly lower expression of proteins associated with antioxidative response pathways (Figure 1B,C). Our results showed significantly reduced relevance abundance of superoxide dismutases (SOD1, SOD2), glutathione peroxidase 1 (GPX1), glutathione S-transferase (GST) family proteins (GSTA3, GSTM1, GSTP1), aldo-keto reductase family 1 member B (AKR1B1), aldehyde dehydrogenase 2 (ALDH2), and methionine sulfoxide reductase A (MSRA). We also conducted pathway enrichment analysis to generate a pathway impact plot using the Gene Set Enrichment Analysis (GSEA) algorithm of MetaboAnalyst 5.0. Protein names were converted to gene IDs to run the GSEA algorithm. The topology analysis showed that colitis impacted glutathione metabolism most significantly, with lesser impact on glycerolipid metabolism, glycolysis/gluconeogenesis, and selenocompound metabolism (Figure 1G and Table S1). The schematic summary of alterations of the antioxidative stress response is shown in Figure 2. Our data collectively indicate that the antioxidative defense is downregulated in DSS-induced colitis, supporting increased ROS and engulfed inflammation in the colon under colitis.

### 3.2. β-Oxidation Pathway Is Downregulated in Colon of Colitis

**Figure 3:**
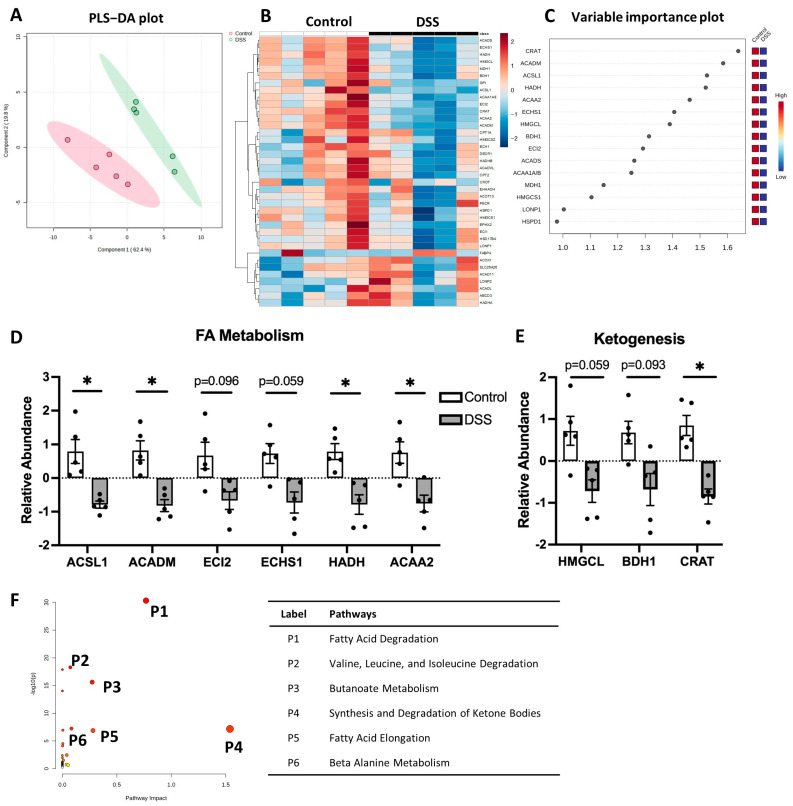
Altered β-oxidation pathway in DSS-induced colitis. (**A**) PLS-DA biplot; (**B**) non-clustered heatmap of correlating protein concentration with individual samples; (**C**) VIP highlighting significantly changed proteins; relative abundance of (**D**) fatty acid (FA) metabolism and (**E**) ketogenesis; (**F**) pathway enrichment impact plot, with color and size of circle indicating pathway impact (larger/more red = higher impact). * *p* < 0.05, DSS vs. Control.

**Figure 4:**
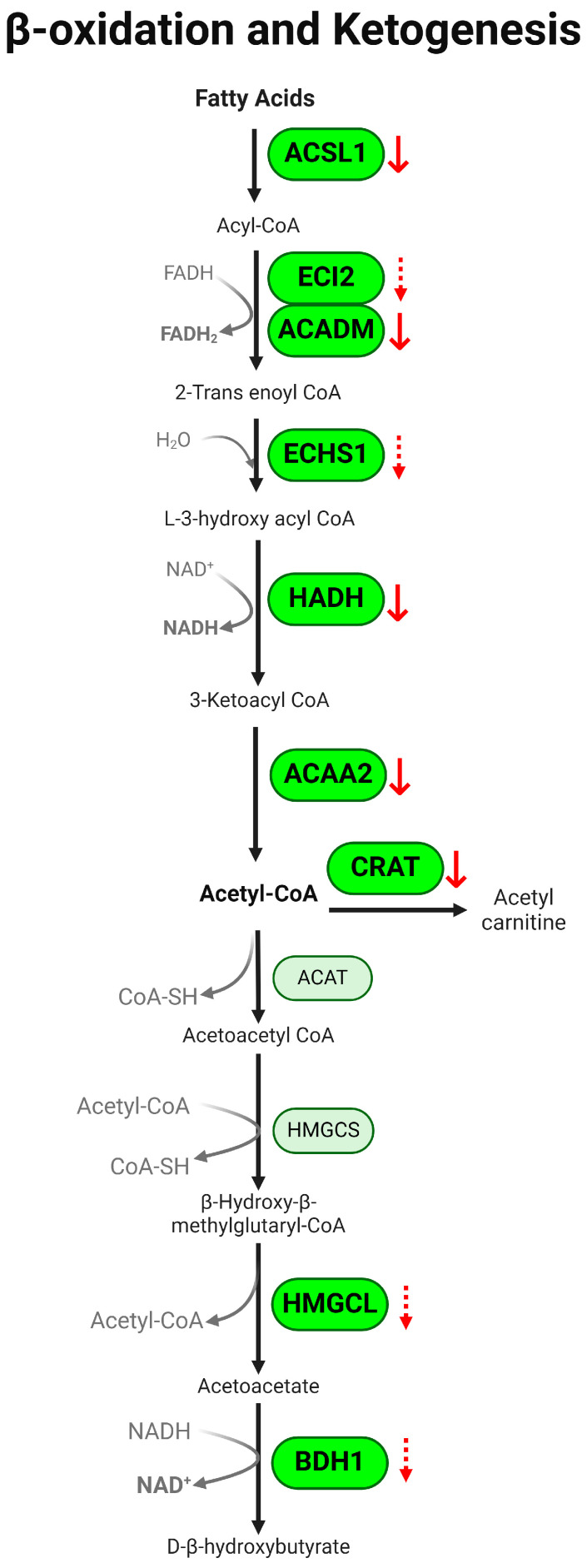
Schematic diagram of β-oxidation and ketogenesis in DSS-induced colitis. Fatty acids are metabolized to acetyl-CoA via β-oxidation, and subsequent ketogenesis converts acetyl-CoA to D-b-hydroxybutyrate. ACSL1, ACADM, HADH, ACAA2, and CRAT were significantly downregulated under colitis. Also, colitis showed a decreasing trend in ECI2, ECHS1, HMFCL, and BDH1 as indicated by the red arrows. dotted ↓ *p* < 0.1, ↓ *p* < 0.05, DSS vs. control. ACSL1: acyl-CoA synthetase; ECI2: enoyl CoA delta isomerase; ACADM: medium-chain acyl-CoA dehydrogenase; ECHS1: enoyl CoA hydratase; HADH: hydroxy-acyl CoA dehydrogenase; ACAA2: acetyl-CoA acyl transferase; CRAT: carnitine acetyltransferase; HMGCL: hydroxy-methylglutaryl lyase; BDH1: hydroxybutyrate dehydrogenase.

Fatty acid β-oxidation is one of the major pathways generating energy metabolizing fatty acid to acetyl-CoA. In our study, 38 proteins associated with the β-oxidation pathway were detected. PLS-DA of DSS-induced colitis mice showed distinct separation from the control group (Figure 3A). The heatmap and VIP score plot showed significantly altered β-oxidation in colitis, and the relative abundance of the identified proteins was significantly reduced in the presence of colitis (Figure 3B,C). Our data identified decreases in long-chain acyl-CoA synthetase (ACSL1), medium-chain acyl-CoA dehydrogenase (ACADM), enoyl CoA delta isomerase (ECI2), enoyl-CoA hydratase (ECHS1), 3-hydroxyacyl CoA dehydrogenase (HADH), and acetyl-CoA acyltransferase 2 (ACAA2) (Figure 3D). We also observed a decreasing trend in proteins regulating the downstream ketogenesis pathway in colitis, including 3-hydroxy-3-methylglutaryl-CoA lyase (HMGCL), 3-hydroxybutyrate dehydrogenase 1 (BDH1), and carnitine acetyltransferase (CRAT) (Figure 3E). Topology analysis using MetaboAnalyst’s GSEA algorithm showed that colitis significantly impacted fatty acid degradation and elongation; the degradation of valine, leucine, and isoleucine; butanoate metabolism; and the synthesis/degradation of ketone bodies (Figure 3F and Table S2). A schematic summary of changes in β-oxidation and ketogenesis in colitis is presented in Figure 4. Together, our results suggest significant downregulation of the FA metabolism through the β-oxidation pathway in colitis.

### 3.3. Glycolysis Pathway Is Downregulated in Colon of Colitis

**Figure 5:**
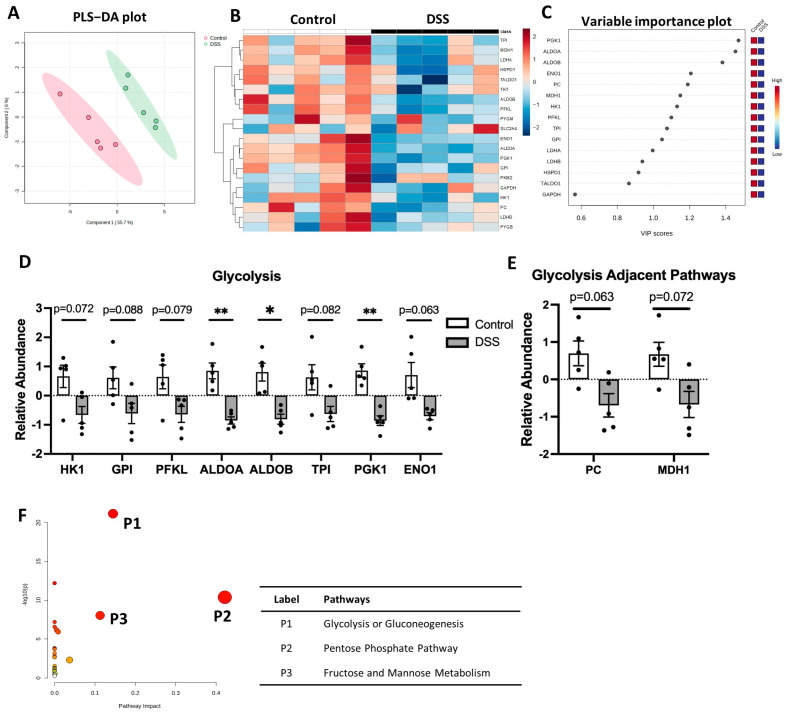
Altered glycolysis pathway in DSS-induced colitis. (**A**) PLS-DA biplot; (**B**) non-clustered heatmap of correlating protein concentration with individual samples; (**C**) VIP score plot highlighting significantly changed proteins; relative abundance of (**D**) glycolysis and (**E**) glycolysis adjacent pathways; (**F**) pathway enrichment impact plot, with color and size of circle indicating pathway impact (larger/more red = higher impact). * *p* < 0.05, ** *p* < 0.01, DSS vs. Control.

**Figure 6:**
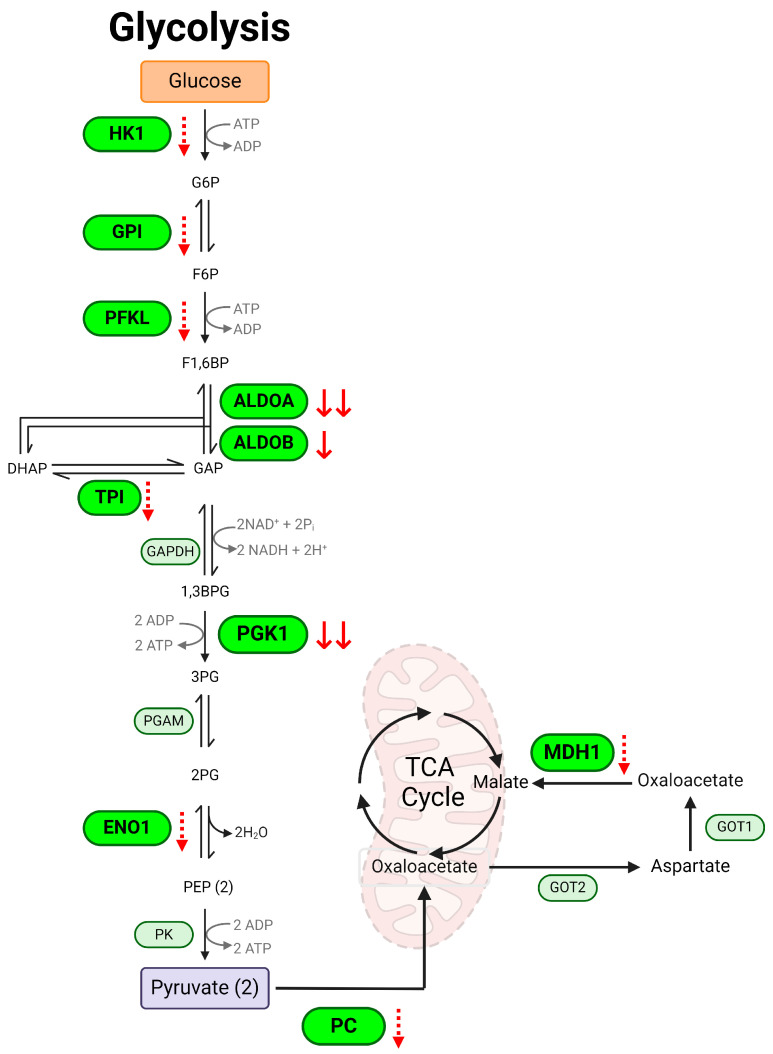
Schematic diagram of glycolysis changes in DSS-induced colitis. Glucose is metabolized to pyruvate via glycolysis. Pyruvate then can be carboxylated to form oxaloacetate, which enters the TCA cycle in the mitochondria. In colitis, the enzymes regulating glycolysis are all reduced, as indicated by red arrows. dotted ↓ *p* < 0.1, ↓ *p* < 0.05, ↓↓ *p* < 0.01, DSS vs. control. HK1: hexokinase; G6P: glucose-6-phosphate; GPI: glucose-6-phosphate isomerase; F6P: fructose-6-phosphate; PFKL: phosphofructokinase; F1,6BP: fructose-1,6-biphosphate; ALDO: aldolase fructose biphosphate; GAP: glyceraldehyde phosphate; DHAP: dihydroxyacetone phosphate; TPI: triosephosphate isomerase; 1,3BPG: 1,3-biphosphoglyceric acid; PGK1: phosphoglycerate kinase; 3PG: 3-phosphoglyceric acid; 2PG: 2-phosphoglyceric acid; ENO1: alpha enolase; PEP: phosphoenolpyruvic acid; PC: pyruvate carboxylase; MDH1: malate dehydrogenase.

Glycolysis is a central anaerobic metabolic pathway producing energy for cell metabolism, and glycolysis is linked to the cellular inflammatory response to meet rapid energy demand upon inflammation [24,25]. We detected 22 proteins regulating glycolysis. We observed significant separation in the PLS-DA plot between DSS-treated colitis and the control mice (Figure 5A). The heatmap and VIP score plot indicated a significant decrease in proteins regulating glycolysis and the glycolysis-linked TCA cycle pathway (Figure 5B,C). We observed a reduced relevant abundance for hexokinase (HK1), glucose-6-phosphateisomerase (GPI), phosphofructokinase (PFKL), fructose 1,6-biphosphate aldolase enzymes (ALDOA, ALDOB), triosephosphate isomerase (TPI), phosphoglycerate kinase 1 (PGK1), and alpha enolase (ENO1) in colitis (Figure 5D). Also, the proteins involved in connecting glycolysis and the TCA cycle showed a decreasing trend, including the pyruvate carboxylase (PC) and cytosolic malate dehydrogenase (MDH1) (Figure 5E). Pathway topology analysis using MetaboAnalyst’s GSEA algorithm indicated that colitis has an impact on glycolysis/gluconeogenesis, the pentose phosphate pathway, and fructose/mannose metabolism (Figure 5F and Table S3). A schematic summary of glycolysis changes in colitis is shown in Figure 6. Overall, our data suggest significant downregulation of glycolysis in experimental colitis.

### 3.4. Tricarboxylic Acid (TCA) Cycle Is Downregulated in Colon of Colitis

**Figure 7:**
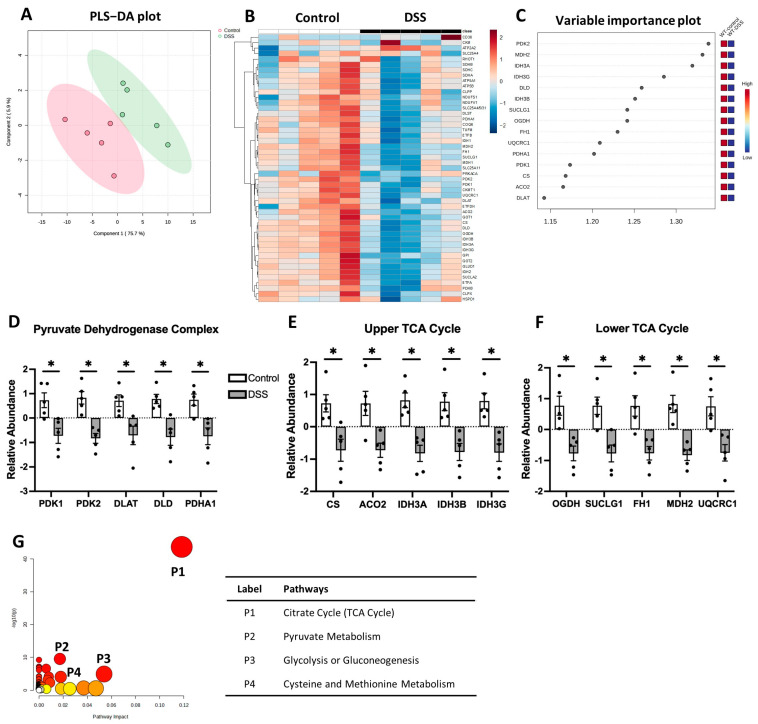
Altered TCA cycle profile in DSS-induced colitis. (**A**) PLS-DA biplot; (**B**) non-clustered heatmap of correlating protein concentration with individual samples; (**C**) VIP score plot highlighting significantly changed proteins; relative abundance of (**D**) pyruvate dehydrogenase complex and (**E**,**F**) the TCA cycle; (**G**) pathway enrichment impact plot, with color and size of circle indicating pathway impact (larger/more red = higher impact). * *p* < 0.05, DSS vs. Control.

**Figure 8:**
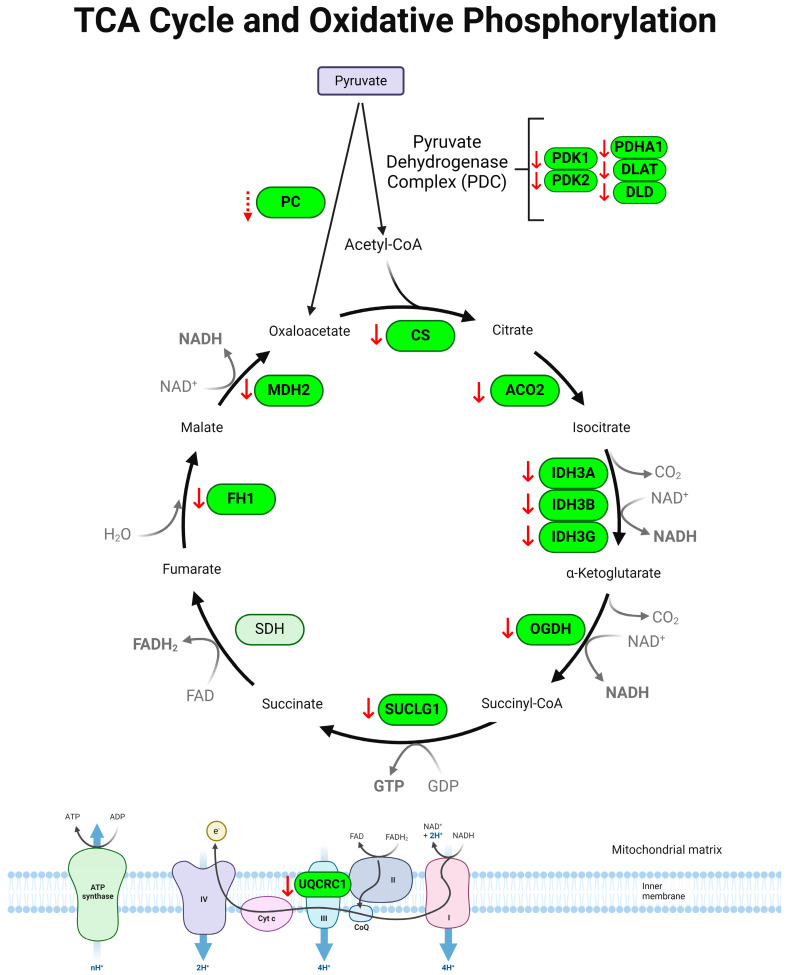
Schematic diagram of TCA cycle re-programming in DSS-induced colitis. Pyruvate enters the mitochondria and converts to acetyl-CoA, which is metabolized via the TCA cycle. Coenzymes NADH and FADH_2_ produced from the TCA cycle then transfer electrons to the ETC to drive OXPHOS and produce ATP. In colitis, most enzymes regulating the TCA cycles were downregulated. UQCRC1, a component of complex III of the ETC, was also downregulated. dotted ↓ *p* < 0.1, ↓ *p* < 0.05, DSS vs. control. PDK: pyruvate dehydrogenase kinase; PDHA1: pyruvate dehydrogenase E1; DLAT: dihydrolipoyl transacetylase; DLD: dihydrolipoamide dehydrogenase; PC: pyruvate carboxylase; CS: citrate synthase; ACO2: aconitase; IDH3: isocitrate dehydrogenase; OGDH: oxoglutarate dehydrogenase; SUCLG1: succinate CoA ligase; FH1: fumarate hydratase; MDH2: malate dehydrogenase; OXPHOS: oxidative phosphorylation; UQCRC1: ubiquinol cytochrome c reductase core protein.

The tricarboxylic acid (TCA) cycle, also known as the citric acid cycle or Krebs cycle, is a signaling hub in mitochondria generating energy by breaking down pyruvate and acetyl-CoA, and regulating various cellular responses [26,27]. In our study, 49 proteins associated with the TCA cycle were detected in the colon with/without colitis. The DSS-induced colitis and control groups showed significant separation in the PLS-DA plot (Figure 7A). The heatmap and VIP score plot showed alterations of TCA cycle proteins in colitis (Figure 7B,C). We observed the robust suppression of TCA cycle-associated proteins including the pyruvate dehydrogenase kinase complex (PDK1, PDK2), dihydrolipoyl transacetylase (DLAT), dihydrolipoyl dehydrogenase (DLD), and pyruvate dehydrogenase E1 alpha subunit (PDHA1) (Figure 7D), which are collectively referred to as the pyruvate dehydrogenase complex (PDC) [28]. Also, significant decreases were observed in citrate synthase (CS), aconitase 2 (ACO2), isocitrate dehydrogenase subunits (IDH3A, IDH3B, IDH3G), 2-oxoglutarate dehydrogenase subunit (OGDH), succinate CoA ligase alpha subunit (SUCLG1), fumarate hydratase (FH1), and malate dehydrogenase 2 (MDH2) (Figure 7E,F). Additionally, ubiquinol cytochrome c reductase core protein 1 (UQCRC1), a component of the mitochondrial electron transport chain (ETC) that acts downstream of the TCA cycle, was significantly reduced (Figure 7F). Pathway topological analysis using the GSEA identified significant impacts of DSS-induced colitis on the TCA cycle, pyruvate metabolism, glycolysis/gluconeogenesis, and cysteine and methionine metabolism (Figure 7G and Table S4). A schematic summary of colitis-associated changes in the TCA cycle is shown in Figure 8. Collectively, our data suggest a significant downregulation of the TCA cycle in the colon of DSS-induced colitis.

## 4. Discussion

Metabolism is essential for all biological processes including development, differentiation, proliferation, and functionality of cells. Metabolism is a dynamic cellular process that generates energy to meet the demand of physiological/pathological conditions, to adapt to the given environment or stimulus [29]. A previous study using RNA sequencing reported that key genes and pathways changed in UC patients; the most upregulated pathways are related to immune activation and cytokine production, and the most downregulated pathways are the mitochondrial TCA cycle and metabolic pathways [30]. Given the significant role of metabolic pathways in immune-cell activation and function, we studied four major metabolic pathways in the colons of mice with experimental colitis using LC/MS proteomics. Our findings revealed that colitis is associated with wide-ranging dysregulation of major metabolic pathways, including oxidative stress, β-oxidation, glycolysis, and the TCA cycle.

IBD is a complex and multifactorial disease. Though most studies in the literature of IBD evaluate only individual pathway. To obtain a more complete and comprehensive understanding of metabolic dysregulation in IBD, we have studied four major metabolic pathways in colitis. Also, many previous studies have used human serum and urine samples due to easy accessibility of bodily fluids [13,31,32,33]. However, several metabolomics studies indicate discrepancies of metabolite levels between blood/urine and colon biopsy samples [13,14,17,18]. It has been suggested that exfoliated cells in feces may serve as a noninvasive method to assess mucosal signatures for early detection/diagnosis of intestinal health [34]. In our study, we analyzed colonic tissue directly and employed a proteomics methodology to assess the alteration of metabolic pathways under colitis. Notably, proteomic profiling of metabolic pathways at the translational level provides more relevant insights into functional outcomes than gene expression. Furthermore, we focused on examining the levels of enzymatic proteins that govern metabolic pathways. The proteomic approach not only provided insights into immediate regulatory events but also offered a better understanding of the mechanistic insights associated with pathogenesis of colitis.

Oxidative stress is a crucial pathological factor that plays an important role in the onset and progression of IBD [23,35,36,37]. ROS promote mucosal epithelium damage, contributing to mucosal barrier dysfunction [38]. Our data showed that colitis significantly downregulated the proteins associated with antioxidant defense in the colon (Figure 1), suggesting a significant accumulation of toxins derived from ROS, including O_2_^•−^, H_2_O_2_, lipid-derived aldehydes, and oxidized methionine. It is in line with our previous report showing significantly increased colonic inflammation accompanied with an increase in disease activity (reduced body weight, increased rectal bleeding, and diarrhea) and inflammatory cytokine expressions in the colon of DSS-induced colitis compared to water control [19]. Of note, a different degree of colitis severity would have differential effects on metabolomic profiles. In our study, we induced ulcerative colitis using 2% DSS, a concentration generally associated with mild induction of ulcerative colitis. The 2% DSS for 7 days of treatment increased the disease activity index (DAI) to approximately half of the maximum score, consistent with the results of our previous report [19]. This indicates that the observed alterations in metabolic pathways in our current study are associated with mild disease activity in ulcerative colitis.

Our current data revealed that the mice with colitis showed decreased enzymes regulating the clearance of detrimental superoxide including O_2_^•−^, H_2_O_2_, lipid-derived aldehydes, and oxidized methionine (Figure 2). Superoxide dismutases (SOD) catalyze the reduction of superoxide radicals (O_2_^•−^) into hydrogen peroxide (H_2_O_2_) [39]. Our study showed that both SOD1 and SOD2 are downregulated in experimental colitis (Figure 1D). This result is in line with other reports that the polymorphism of SOD1 is associated with increased risk of UC [40], and the polymorphism of SOD2 affects UC onset [41], underscoring the protective role of SOD in UC. On the other hand, one study has reported that SOD levels are elevated in IBD patients, whereas the levels can be restored to normal in remission [42]. However, the low enzymatic activity of SOD is associated with the active stage of IBD [42,43], which suggests that SOD activity is critical in pathogenesis of UC. Our finding is further supported by the evidence that SOD1 deficiency exacerbates DSS-induced colitis and SOD treatment significantly attenuates colonic inflammation in experimental colitis [44,45].

Glutathione peroxidases (GPX) are selenoproteins with antioxidant/anti-inflammatory functions. They protect cell damage from peroxides by catalyzing glutathione (GSH) into an oxidized form (glutathione disulfide (GSSH)), reducing H_2_O_2_ into H_2_O. Our data indicated a notable decrease in the GPX1 level in colitis (Figure 1E). This finding is consistent with the other reports showing an association of GPX1 and IBD onset [46], and a double knockout of Gpx1 and Gpx2 genes triggers colitis [46]. Given the study showing the reduced enzymatic activity of GPX and GSH in SOD1 deficiency in a DSS-induced colitis model [45], we cannot exclude the possibility that a lower expression of GPX1 in our study is the result of reduced SOD in colitis.

GST and ALDH2 are the enzymes that reduce lipid-derived aldehydes (HNE). Our results revealed that the levels of these enzymes are all reduced in colitis (Figure 1E). Glutathione transferases (GST) are a group of enzymes consisting of four classes (alpha, mu, pi, and theta), which conjugate with glutathione to regulate scavenging lipid-derived aldehydes (HNE). We found that colitis significantly decreased GST classes alpha, mu, and pi (GSTA3, GSTM1, GSTP1) (Figure 1E). Our data are supported by previous reports showing low GSTM in the early age onset of UC [47,48,49], whereas limited studies can be found in regard of GSTA and GSTP in IBD. Similar to GST, aldehyde dehydrogenases (ALDH) have a key role in converting aldehydes to harmless HNA. Our result of reduced ALDH2 in colitis could potentially be explained by the reduced presence of the ALDH^+^ macrophage and dendritic cells in the colon of UC [50], considering that inflammatory myeloid cells are largely recruited to the lamina propria in UC [51]. On the other hand, an increased ALDH level in colon stem cells has been shown to mediate the transition from colitis to cancer, suggesting the differential role of ALDH in different cell types [52].

MSRA is a key antioxidant enzyme that catalyzes the reduction of methionine S-sulfoxide. Our study showed that colitis decreased MSRA in the colon (Figure 1F). While limited studies have investigated whether MSRA is involved in UC pathogenesis, it has been shown that the deletion of MSRA induces a higher susceptibility to LPS-induced lethal shock [53]. In the same study, bone marrow-derived macrophages with MSRA deficiency showed elevated ROS levels along with higher NF-kB nuclear translocation. This is in line with our results, suggesting that lower MSRA expression likely contributes to high ROS levels, which in turn increases vulnerability to colitis. Together, our findings suggest that antioxidative defense pathways are significantly downregulated in colitis. This failure of antioxidative defense contributes to increased ROS levels, which strongly indicates colonic inflammation.

Ulcerative colitis is often described as an energy-deficient state accompanied by oxidative stress [54]. β-oxidation is an important energy-generating pathway. The association between IBD and fatty acid β-oxidation has been reported [54,55]. Our data showed that the enzymes ACSL1 and ACADM, which break down long-chain fatty acids and medium-chain fatty acids, respectively, were significantly decreased in colitis. One study reported that ACSL1 was upregulated in the colons of UC patients, although only mRNA levels were tested in the study [56]. It was suggested that the primary function of ACSL1 is to promote triacylglycerols and de-novo synthesis of phospholipids. The alterations of ACSL1 in β-oxidation could be a compensatory mechanism for disease progression [57]. β-oxidation of short-chain fatty acids (SCFAs), especially butyrate, is a preferred energy source in epithelial cells that govern the maintenance of mucosal homeostasis [58,59]. Our results showed that the enzymes regulating β-oxidation were significantly downregulated in the colon of colitis (Figure 3 and Figure 4), suggesting a contribution of diminished β-oxidation of SCFAs in UC. Our findings are in line with previous reports indicating decreased oxidation and uptake of butyrate in IBD patients [54,55,60]. Additionally, our results are in line with the report that the deletion of β-oxidation in the epithelium develops ulcerative colitis [61]. ECHS1 is associated with butyrate metabolism. Notably, our study showed a reduction in ECHS1, which is consistent with the downregulation of the gene encoding ECHS1 observed in the inflamed mucosa of UC patients [55,62]. These findings collectively suggest that the diminished capacity of the colon to utilize SCFAs for energy generation contributes to mucosal dysbiosis and hinders epithelial regeneration in UC.

Glycolysis is one of the major metabolic pathways which is the main source of energy for cells. Glycolysis converts glucose to pyruvate. The end products of this process enter the TCA cycle to be fully metabolized, generating NADH and FADH_2_ to promote oxidative phosphorylation (OXPHOS). Our study shows a reduced expression of the regulatory enzymes of glycolysis in the colon under colitis (Figure 5 and Figure 6). Our finding is consistent with another study that demonstrated reduced levels of glycolysis-derived metabolites in the colon biopsies of IBD patients [17]. Specifically, our results revealed decreased levels of glycolytic enzymes, including HK1, GPI, PFKL, ALDOA, and ALDOB in colitis. This aligns with the reduced levels of myoinositol and glycerol-phosphate identified in IBD, both of which are products governed by the aforementioned enzymes that were reduced in our study. Differential outcomes in gene enrichment analysis of glycolysis have been reported in IBD by others [63,64]. Our study specifically assessed the translation of key enzymes, in contrast to prior studies that examined glycolysis largely at the mRNA level. It is possible that the downregulation of glycolysis at the translational level leads to compensatory stimulation of mRNA transcription to meet the cellular metabolic demands arising from the reduced glycolytic rate. Collectively, our study showed a reduced expression of glycolytic enzymes, suggesting the downregulation of glycolysis.

The TCA cycle is a crucial metabolic pathway in the mitochondria. It plays an essential role in ATP generation. Our results revealed that the majority of enzymes regulating the TCA cycle were significantly suppressed in experimental colitis (Figure 7 and Figure 8), suggesting a significant suppression of the TCA cycle in colitis. Our results are in line with reports showing decreased TCA cycle-related metabolites in IBD, such as citrate, isocitrate, a-ketoglutarate, succinate, fumarate, and malate in UC [13,16,17,18,31,32,33,65,66,67]. The fuels for the operation of the TCA cycle are acetyl-CoA and pyruvate, which are the end products of β-oxidation and glycolysis. Given that our results show a simultaneous downregulation of β-oxidation and glycolysis (Figure 3, Figure 4, Figure 5 and Figure 7), we expect that the low availability of acetyl-CoA and pyruvate for the TCA cycle likely contributes to the dysregulation of the TCA cycle in colitis. Further studies utilizing gain-of-function or loss-of-function in animal and/or cell models to target the specific pathways would provide more insights into the necessity and sufficiency of the regulators, further advancing the understanding of the potential interplay among these dysregulated metabolic pathways in IBD.

The TCA cycle produces electron carriers, such as NADH and FADH_2_, which deliver electrons into the electron transport chain (ETC), generating a proton gradient across the inner mitochondrial membrane [27,68]. This proton gradient leads the ATP synthase complex to generate ATP. The energy production process is known as OXPHOS. In our study, we observed a significant reduction in UQCRC1 in colitis, which is a subunit of mitochondrial complex III in ETC (Figure 7 and Figure 8). Our result is similar to previous studies showing the reduced gene expression of *Uqcrc1* in UC and colorectal cancer [69,70]. Given the intricate connection of the TCA cycle and ETC in mitochondria, TCA cycle dysregulation and mitochondrial dysfunction may exacerbate colitis [30]. Indeed, our findings indicated dysregulation in the resolution of oxidative stress in the mice with colitis (Figure 1 and Figure 3), suggesting the involvement of a defective TCA cycle and a dysfunctional ETC in the mitochondria in the pathogenesis of colitis. Collectively, our findings indicate that dysregulation of the TCA cycle and mitochondrial ETC is involved in the pathogenesis of colitis.

Our current study helps to better understand metabolic pathways and their dysregulation in the colon under colitis. However, we are aware there are several limitations with our current study. CD and UC are characterized by both shared and distinct pathogenic features. While DSS-induced colitis serves as a well-established model for UC, it does not replicate the characteristics of CD. Hence, our findings are specifically relevant to ulcerative colitis. Also, our study conducted targeted proteomics, specifically focusing on four major metabolic pathways. However, it is important to note that other metabolic pathways such as amino acid metabolism, may also be involved in the pathogenesis of colitis [13,16]. Therefore, future study utilizing untargeted analysis would be beneficial in providing more comprehensive insights. Sexual dimorphism between males and females extends to various aspects of metabolism. Studies have shown that the severity and progression of inflammatory responses differ between male and female subjects; there are higher incidences and remission rates of colitis in males compared to females after age 45 [71,72,73,74]. Hormonal variations, particularly estrogen levels in females, may influence the metabolic pathways involved in inflammation and tissue repair in the colon during colitis [75,76,77]. Our experiments were conducted with male mice. For further study, it is essential to also examine the metabolic pathways in female mice under colitis.

Notably, the colon lamina propria serves as a primary reservoir of a large body of infiltrated immune cells upon intestinal inflammation, which contributes significantly to the pathogenesis and progression of the disease [78,79]. We and others have previously reported that immune cells undergo a functional change in their microenvironment by shifting their metabolism from OXPHOS to glycolysis to meet the high demand of energy [80,81,82,83,84]. The current study used colon tissue, which includes multiple cell types from all layers of the colon (for example, the epithelium, lamina propria, and muscularis mucosa). The detected proteomics profile reflects this variety of cell types. In our current study, we were unable to differentiate the metabolic pathways in specific cell types in colitis. Further exploration using cell-specific deletion models would advance our understanding of the cell-specific regulation of the key metabolic pathways in IBD.

## 5. Conclusions

This study delved into the alterations in four major metabolic pathways including oxidative stress, β-oxidation, glycolysis, and the TCA cycle, in experimental colitis. The findings underscore the significance of energy metabolism in the pathogenesis of IBD, and specifically highlight the pervasive dysfunction of metabolic pathways in colitis. Further investigation of metabolic programming in specific cells, such as immune or epithelial cells, will further advance the understanding of tissue/cell specificity and the underlying mechanisms of IBD, leading to novel therapeutic interventions.

## Data Availability

The raw data supporting the conclusions of this article will be made available by the authors on request.

## Supplementary Materials

The following supporting information can be downloaded at: https://www.mdpi.com/article/10.3390/metabo14040194/s1, Table S1: Oxidative stress-related pathways in DSS-induced colitis, Table S2: β-oxidation-related pathways in DSS-induced colitis, Table S3: Glycolysis-related pathways in DSS-induced colitis, Table S4: TCA cycle-related pathways in DSS-induced colitis.
