# Supplementary material for: Colonic Dysregulation of Major Metabolic Pathways in Experimental Ulcerative Colitis
Source: Metabolites. 2024 Mar 29;14(4):194. doi: 10.3390/metabo14040194 (PMC11052278; doi:10.3390/metabo14040194)
Supplement: Supplementary file 1 [file metabolites-14-00194-s001.zip › metabolites-2808336-supplementary.pdf]

Supplementary information

Colonic Dysregulation of Major Metabolic Pathways in Experimental Ulcerative Colitis

Table S1. Oxidative stress-related pathways in DSS-induced colitis.

Label	Pathways	Total	Expected	Hits	Raw p	-log10(p)	Holm adjust	FDR	Impact
P1	Glutathione metabolism	65	0.17339	5	6.3114E-07	6.1999	0.00020575	0.00010319	0.064815
P2	Glycerolipid metabolism	61	0.16272	2	0.011322	1.9461	1	0.2603	0.11438
P3	Glycolysis or Gluconeogenesis	66	0.17606	2	0.013164	1.8806	1	0.2603	0.053922
P4	Selenocompound metabolism	17	0.045349	1	0.044434	1.3523	1	0.5812	0.021978
P5	beta-Alanine metabolism	32	0.085363	1	0.082067	1.0858	1	0.70621	0.090909
P6	Pentose and glucuronate interconversions	34	0.090698	1	0.086977	1.0606	1	0.71103	0.019231
P7	Amino sugar and nucleotide sugar metabolism	49	0.13071	1	0.123	0.91008	1	0.91093	0.036998
-	Fatty acid degradation	50	0.13338	1	0.12536	0.90185	1	0.91093	0.00026441
-	Arginine and proline metabolism	50	0.13338	1	0.12536	0.90185	1	0.91093	0.0094467
-	Valine, leucine and isoleucine degradation	56	0.14939	1	0.13935	0.8559	1	0.9695	0.0034368

Pathway analysis was conducted using Fischer's exact test for enrichment analysis and betweenness centrality for topology analysis. **Total:** the total number of proteins in the pathway; **Expected**: the number of proteins in the pathway that would be expected to be altered by chance alone; Hits: the number of proteins in the pathway that were altered according to our data; **Raw p**: raw p-value; **-log10(p):** the negative logarithm base 10 of the raw p-value, which is used as the y-axis on the significance vs. impact plot; **Holm adjust**: p-values adjusted using the Holm-Bonferroni method; **FDR**: false discovery rate-adjusted p-values; **Impact**: the relative impact on the pathway from enrichment and topology analysis.

Table S2. β-oxidation-related pathways in DSS-induced colitis.

Label	Pathways	Total	Expected	Hits	Raw p	-log10(p)	Holm adjust	FDR	Impact
P1	Fatty acid degradation	50	0.19137	17	4.8716E-31	30.312	1.593E-28	1.593E-28	0.76776
P2	Valine, leucine and isoleucine degradation	56	0.21434	12	5.1303E-19	18.29	1.6725E-16	8.3881E-17	0.073129
P3	Butanoate metabolism	27	0.10334	9	2.3909E-16	15.621	7.7465E-14	1.9545E-14	0.2735
P4	Synthesis and degradation of ketone bodies	11	0.042102	4	5.7589E-08	7.2397	1.8544E-05	3.1106E-06	0.083333
P5	Fatty acid elongation	29	0.111	5	6.6587E-08	7.1766	2.1375E-05	3.1106E-06	1.5384
P6	beta-Alanine metabolism	32	0.12248	5	1.12E-07	6.9508	3.584E-05	4.5779E-06	0.004329
-	Propanoate metabolism	33	0.1263	5	1.3164E-07	6.8806	4.1993E-05	4.7829E-06	0.2808
-	Tryptophan metabolism	48	0.18372	4	3.0734E-05	4.5124	0.0097735	0.001005	0.004065
-	Lysine degradation	61	0.23347	4	7.9592E-05	4.0991	0.025231	0.0023661	0.0019763
-	Terpenoid backbone biosynthesis	23	0.088031	2	0.003418	2.4662	1	0.093141	0.040441

Pathway analysis was conducted using Fischer's exact test for enrichment analysis and betweenness centrality for topology analysis. **Total:** the total number of proteins in the pathway; **Expected**: the number of proteins in the pathway that would be expected to be altered by chance alone; Hits: the number of proteins in the pathway that were altered according to our data; **Raw p**: raw p-value; **-log10(p):** the negative logarithm base 10 of the raw p-value, which is used as the y-axis on the significance vs. impact plot; **Holm adjust**: p-values adjusted using the Holm-Bonferroni method; **FDR**: false discovery rate-adjusted p-values; **Impact**: the relative impact on the pathway from enrichment and topology analysis.

Table S3. Glycolysis-related pathways in DSS-induced colitis.

Label	Pathways	Total	Expected	Hits	Raw p	-log10(p)	Holm adjust	FDR	Impact
**P1**	Glycolysis or Gluconeogenesis	66	0.14544	12	6.8117E-22	21.167	2.2274E-19	2.2274E-19	0.14468
**P2**	Pentose phosphate pathway	32	0.070517	6	4.1736E-11	10.379	1.3564E-08	4.5493E-09	0.42139
**P3**	Fructose and mannose metabolism	35	0.077128	5	9.1385E-09	8.0391	2.9609E-06	7.4707E-07	0.11275
**-**	Starch and sucrose metabolism	33	0.072721	4	6.6198E-07	6.1792	0.00021249	3.0924E-05	0.0047619
**-**	Pyruvate metabolism	38	0.083739	4	1.1858E-06	5.926	0.00037947	4.8471E-05	0.008658
**-**	Amino sugar and nucleotide sugar metabolism	49	0.10798	2	0.005087	2.2935	1	0.10397	0.036998
**-**	Purine metabolism	136	0.2997	1	0.26096	0.58342	1	1	0.0016193

Pathway analysis was conducted using Fischer's exact test for enrichment analysis and betweenness centrality for topology analysis. **Total:** the total number of proteins in the pathway; **Expected**: the number of proteins in the pathway that would be expected to be altered by chance alone; Hits: the number of proteins in the pathway that were altered according to our data; **Raw p**: raw p-value; **-log10(p):** the negative logarithm base 10 of the raw p-value, which is used as the y-axis on the significance vs. impact plot; **Holm adjust**: p-values adjusted using the Holm-Bonferroni method; **FDR**: false discovery rate-adjusted p-values; **Impact**: the relative impact on the pathway from enrichment and topology analysis.

Table S4. TCA cycle-related pathways in DSS-induced colitis.

Label	Pathways	Total	Expected	Hits	Raw p	-log10(p)	Holm adjust	FDR	Impact
P1	TCA cycle	32	0.14846	21	1.9496E-44	43.71	6.3753E-42	6.3753E-42	0.11825
P2	Pyruvate metabolism	38	0.17629	7	3.0241E-10	9.5194	9.8586E-08	4.9444E-08	0.017316
P3	Glycolysis or Gluconeogenesis	66	0.30619	5	1.2057E-05	4.9188	0.0038462	0.00043807	0.053922
P4	Cysteine and methionine metabolism	52	0.24124	4	9.158E-05	4.0382	0.028848	0.0023036	0.018018
-	Glyoxylate and dicarboxylate metabolism	31	0.14382	5	2.5811E-07	6.5882	8.2855E-05	1.2058E-05	0.0059289
-	Arginine and proline metabolism	50	0.23196	4	7.8428E-05	4.1055	0.024862	0.0023036	0.0080972
-	Alanine, aspartate and glutamate metabolism	38	0.17629	3	0.00069736	3.1565	0.21758	0.014252	0.0070565
-	Phenylalanine metabolism	23	0.1067	2	0.0049924	2.3017	1	0.085923	0.0090909
-	Tyrosine metabolism	40	0.18557	2	0.014647	1.8343	1	0.21148	0.0010101
-	Calcium signaling pathway	192	0.89074	3	0.058872	1.2301	1	0.61401	0.0026596

Pathway analysis was conducted using Fischer's exact test for enrichment analysis and betweenness centrality for topology analysis. **Total:** the total number of proteins in the pathway; **Expected**: the number of proteins in the pathway that would be expected to be altered by chance alone; Hits: the number of proteins in the pathway that were altered according to our data; **Raw p**: raw p-value; **-log10(p):** the negative logarithm base 10 of the raw p-value, which is used as the y-axis on the significance vs. impact plot; **Holm adjust**: p-values adjusted using the Holm-Bonferroni method; **FDR**: false discovery rate-adjusted p-values; **Impact**: the relative impact on the pathway from enrichment and topology analysis.
